# Bombesin-Tethered Reactive Oxygen Species (ROS)-Responsive Nanoparticles for Monomethyl Auristatin F (MMAF) Delivery

**DOI:** 10.3390/bioengineering8040043

**Published:** 2021-03-29

**Authors:** Jihoon Kim, Jee Seon Kim, Kyung Hyun Min, Young-Hwa Kim, Xiaoyuan Chen

**Affiliations:** 1 Parker H. Petit Institute for Bioengineering and Bioscience, Georgia Institute of Technology, 315 Ferst Dr NW, Atlanta, GA 30332, USA; 2 LG Chem, Seoul 07336, Korea; jeeseon.kim.80@gmail.com; 3 Department of Pharmacy, School of Pharmacy, Jeonbuk National University, Jeonju 54896, Korea; khmin1492@jbnu.ac.kr; 4 Mechanical Design Engineering, Jeonbuk National University, Jeonju 54896, Korea; younghwa.km@gmail.com; 5 Yong Loo Lin School of Medicine and Faculty of Engineering, National University of Singapore, Singapore 117597, Singapore

**Keywords:** auristatin drugs, MMAF, bombesin, reactive oxygen species-responsive, drug delivery

## Abstract

Dolastatin derivatives, represented by monomethylauristatin E (MMAE), have been translated in clinic with a form of antibody–drug conjugate; however, their potential in nanoparticle systems has not been well established due to the potential risk of immature release of extremely high cytotoxic dolastatin drugs during blood circulation. Herein, we rationally propose monomethylauristatin F (MMAF), a dolastatin-derived, loaded nanoparticle system composed of bombesin (BBN)-tethered ROS-responsive micelle system (BBN-PEG-PPADT) to achieve efficient anticancer therapy with targeted and efficient delivery of MMAF. The developed MMAF-loaded BBN-PEG-PPADT micelles (MMAF@BBN-PEG-PPADT) exhibited improved cellular uptake via interactions between BBN and gastrin-releasing peptide receptors on the cancer cells and the intracellular burst release of MMAF, owing to the ROS-responsive disruption, which allowed the efficient anticancer effects of MMAF in vitro. This study suggests the potential of nanoparticle systems in the delivery of dolastatin drugs.

## 1. Introduction

Since dolastatins were first derived from the mollusc *Dolabella auricularia* and their anticancer activities were demonstrated, there have been tremendous efforts to investigate their mechanism and clinical potential. Soblidotin (TZT-1027), dolastatin-10, monomethylauristatin E (MMAE), and monomethylauristatin F (MMAF) are representative examples of dolastatin derivatives that are known to exert anticancer effects by inhibiting tubulin polymerization and inducing apoptosis and intratumoral vascular damage [1,2,3,4,5,6]. In particular, these drugs have 100–1000 times more potency than doxorubicin, and have attracted much attention in commercial and practical clinical applications by investigating the library of their derivatives and developing formulations.

In 2011, Adcetris was approved by the US Food and Drug Administration (FDA) for the treatments of Hodgkin’s lymphoma [7,8]. Adcetris is an antibody–drug conjugate (ADC) that comprises MMAE, a self-immolative linker, an enzymatic cleavable linker, and a tumor-targeting antibody [4,7,8,9]. As contrasted with free MMAE, which cannot be used as a drug itself due to its nonspecific high cytotoxicity, ADC formulation of MMAE not only enables MMAE to hide its toxicity during blood circulation, but also facilitates exerting its therapeutic effects via targeted delivery with antibody-receptor-specific endocytosis and metabolism with intracellular enzymatic cleavage. The successful clinical translation of MMAE with ADC forms have inspired many researchers and pharmaceutical companies to ramp up the development of antibody–MMAE conjugates [10].

In addition to MMAE, MMAF is also one of the important drugs that have been widely employed in developing ADCs. There have been numerous studies that compared the anticancer potency of MMAE and MMAF in their free or ADC forms [5,11,12,13]. Free MMAF has much lower cytotoxic activity than free MMAE in vitro; however, the modification of MMAF from the carboxylic acid group to the methoxy group facilitates higher cytotoxic effects than MMAE. Therefore, it is suggested that the lower activity of MMAF compared to MMAE presumably owes to the charged *C*-terminal phenylalanine group that impairs intracellular access [5]. Interestingly, the anticancer effects of MMAF ADC are higher than those of MMAE ADC in vitro [11], whereas the opposite results are shown in vivo [12,13]. These in vitro and in vivo results collectively imply that the MMAF can exhibit a higher therapeutic index than MMAE, if MMAF can be efficiently internalized into the cells.

The deconjugation between antibody and payload that comprise drug, self-immolative linker, and enzyme-cleavable linker with maleimide groups has been seen as one of the reasons why experimental results of antibody–MMAF in vivo is not correlated with those in vitro [14,15]. Allen Ebens’ groups reported that the linker between the antibody and the drugs are cleaved by an unknown mechanism during blood circulation [14]. In particular, Cong et al. of Pfizer Inc. found that a majority of the payloads are deconjugated from the antibody during blood circulation, and then the resultant maleimide groups of payloads bind to plasma components, such as albumin. These results cast doubts on the traditional mechanism of ADC. Furthermore, they carefully imply that the low therapeutic efficiency of antibody–MMAF may be ascribed to the low internalization of payloads because the MMAF may be delivered to the target cells not mainly via antibody-receptor endocytosis, but via an unknown other mechanism.

In addition to the aforementioned unclear mechanisms, ADC formulation has several problems. In developing stages, it is difficult to select antibody/drug pairs and linkers, which significantly affect the selective targeting and efficient intratumoral release of the free drug. The system is cost-ineffective because it requires complex chemical reactions with low yields, and only 4–8 drugs can be delivered with one expensive antibody. In particular, this system heavily depends on antigen–antibody-specific endocytosis, although this pathway is not clear, which only allows its use for cancers with specific ligands.

**Scheme 1:**
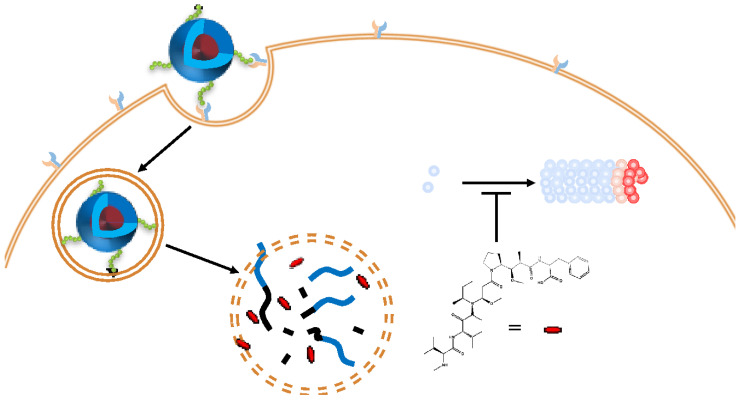
Schematic actions of MMAF delivery systems with active targeting ability and ROS-responsiveness. The nanoparticles are efficiently internalized into the tumor cells. Intracellular ROS facilitates the disruption of the nanoparticles, which release MMAF to exert the anticancer effects by inhibiting tubulin polymerization.

Herein, we developed ADC-mimicking nanoparticle-based systems for efficient delivery of auristatin drugs to address the aforementioned issues (Scheme 1). We have developed an active targeting peptide-tethered, reactive oxygen species (ROS)-responsive micelle system composed of bombesin-tethered poly(ethylene glycol)-*block*-poly(1,4-phenyleneacetone dimethylene thioketal) (PEG-*b*-PPADT). The hydrophobic interactions between auristatin drugs and PPADT facilitates the physical loading of the drugs into the micellar core, which allows the loading a number of drugs into the delivery systems while avoiding the dissipation of expensive auristatin drugs. In particular, MMAF was selected for this delivery system rather than MMAE because the premature release of MMAE before targeted delivery is at potential risk for inducing severe side effects, as contrasted with MMAF, with lower cytotoxic activity in its free form. In addition, the conjugation of bombesin (BBN) peptides on the surface of nanoparticles allows not only passive targeting via enhanced permeation and retention (EPR) effects [15,16], but also active targeting to human gastrin-releasing peptide (GRP) receptor overexpressing cancers, such as prostate, breast, gastrointestinal and small cell lung cancers [17,18]. After the nanoparticles are efficiently internalized to the target cells, the high level of ROS in cancer cells are expected to disrupt the micelles to induce the burst MMAF release [19,20].

## 2. Experimental Procedures

### 2.1. Materials

The 1,4-Benzenedimethanethiol (BDT), 2,2-dimethoxypropane (DMP), and *p*-Toluenesulfonic acid (PTSA) were obtained from Sigma-Aldrich (St. Louis, MO, USA). The mPEG5K-MAL and MAL-PEG5K-NH_2_ were purchased from Biochempeg (Watertown, MA, USA). Monomethyl auristatin F (MMAF) was obtained from DC Chemicals (Pudong, Shanghai, China). Cys-Aca-BBN(7-14)NH_2_ peptide was purchased from CS Bio Co. (Menlo Park, CA, USA). Succinimidyl 4-(*N*-maleimidomethyl)cyclohexane-1-carboxylate (SMCC), Dulbecco’s modified Eagle’s medium (DMEM), Dulbecco’s phosphate-buffered saline (PBS), and fetal bovine serum (FBS) were obtained from Gibco BRL (Grand Island, NY, USA). AlamarBlue reagent was purchased from Thermo Fisher Scientific (Waltham, MA, USA).

### 2.2. Synthesis of Polymers

ROS-responsive PPADT block was synthesized according to reported methods [21,22]. Briefly, DMP (983 mg, 8.0 mmol) was added to anhydrous toluene (50 mL) in a two-neck flask (250 mL) equipped with a distillation head. TSA (4.57 mg, 24 μmol) in anhydrous ethyl acetate (5 mL) was added to the solution and magnetically stirred for 1 h at room temperature. BDT (1.36 g, 8.0 mmol) was added to the mixture, which was then heated to 95 °C. After 30 min, DMP (983 mg, 8.0 mmol) and TSA (4.57 mg, 24 μmol) in anhydrous ethyl acetate (5 mL) solution were dissolved in anhydrous toluene (20 mL) and added to the reaction solution at a rate of about 30 μL min^−1^. The reaction was allowed to stir for 24 h more. The crude product was precipitated in cold *n*-hexane to obtain a brown sticky polymer.

To the solution of PPADT in DCM (20 mL, 5 mg mL^−1^), mPEG5K-MAL (50 mg, 10 μmol) or MAL-PEG5K-NH_2_ (50 mg, 10 μmol) was added and stirred for 2 h at room temperature. The reaction mixture was evaporated and redissolved in 20 mL of degassed deionized water, and was filtered to remove unreacted PPADT. The filtrate was further purified by the three 8000× rpm centrifugations with MWCO 10,000 Amicon tubes, followed by freeze-drying. The successful synthesis of polymers was confirmed by ^1^H nuclear magnetic resonance spectroscopy (^1^H NMR) spectra using a Bruker Advance 300 MHz FT-NMR with deuterium chloroform (CDCl_3_) as a solvent.

BBN-modified PEG-PPADT polymers (BBN-PEG-PPADT) were synthesized by the reactions with NH_2_-PEG-PPADT (30 mg, Mn ≈12,300), Cys-Aca-BBN(7-14)NH_2_ peptides (6.4 mg, 4.9 μmol), and SMCC (1.6 mg, 4.9 μmol) in 2 mL of DMF for 16 h, followed by purification process with three 8000× rpm centrifugations with MWCO 10,000 Amicon tubes and freeze-drying.

FITC-modified PEG-PPADT polymers (BBN-PEG-PPADT) for confocal laser scanning microscopy (CLSM) experiments were synthesized by the reactions with NH_2_-PEG-PPADT (30 mg, Mn ≈ 12,300) and FITC (9.5 mg, 24.4 μmol) in 15 mL of PBS for 16 h, followed by purification process with three 8000× rpm centrifugations with MWCO 10,000 Amicon tubes and freeze-drying.

### 2.3. Preparation of Nanoparticles

Nanoparticles were prepared by a simple emulsification and solvent-evaporation method. Briefly, 10 mg of polymers and 1 mg of MMAF were dissolved in 1 mL dichloromethane (DCM), then dropped into 10 mL deionized water (D.W.) for 3 min. After 5 min vigorous stirring and 1 min vortex, the suspension was ultrasonicated (20 kHz, 91 W, 5 min), and the DCM was removed by rotary evaporation. The nanoparticle solutions were kept in a 4 °C refrigerator before their use. The compositions for each nanoparticle were as following: 10 mg of mPEG-PPADT for the mPEG-PPADT nanoparticles; 8 mg of mPEG-PPADT and 2 mg of BBN-PEG-PPADT for the BBN-PEG-PPADT nanoparticles; 9 mg of mPEG-PPADT and 1 mg of FITC-PEG-PPADT for the CLSM study of the mPEG-PPADT nanoparticles; and 7 mg of mPEG-PPADT, 1 mg of FITC-PEG-PPADT, and 2 mg of BBN-PEG-PPADT for the CLSM study of the BBN-PEG-PPADT nanoparticles.

### 2.4. High-Performance Liquid Chromatography (HPLC) Conditions

An Ultimate 3000 HPLC system with an Ultimate 3000 photodiode array detector (PDA) using a C18 HPLC column (DIONEX C18, 5 μm, 120 Å, 4.6 mm × 250 mm) was used to quantify the amounts of MMAF. The HPLC condition followed [23]; constant 30% A (0.1% TFA in acetonitrile) and 70% B (0.1% TFA in water) for 3 min, linear gradient to 45% A from 3 min to 17 min, linear gradient to 95% A from 18 min to 19 min, constant 95% from 19 min to 21 min, linear gradient to 30% A from 21 min to 22 min, and constant 30% A from 22 min to 25 min with a flow rate of 0.5 mL/min at 80 °C at 214 nm. This method was exploited to take a standard curve of the MMAF, which was further used to measure the drug-loading contents, drug-loading efficiency, and drug-release profile.

### 2.5. Confirmation of ROS-Responsiveness of the Nanoparticles

The size and morphology of the nanoparticles before and after exposure to ROS were observed and measured by transmission electron microscopy (TEM) on a Tecnai T12 microscope (FEI Company, Hillsboro, OR, USA), and dynamic light scattering (DLS) (SZ-100, HORIBA, Kyoto, Japan). According to previously reported methods [24,25], the ROS condition was prepared by generating hydroxyl radicals via mixing 500 μL of nanoparticle-containing solution with 100 μL 16 mM CuCl_2_, 400 μL DW, and 20.34 μL H_2_O_2_.

### 2.6. Drug-Release Test

A total of 680 μL of MMAF loaded BBN-PEG-PPADT micelles (MMAF@BBN-PEG-PPADT) nanoparticle-containing solution was mixed with 340 μL 16 mM CuCl_2_, 2380 μL PBS, and 69.2 μL H_2_O_2_ and then poured into the 3 mL Slide-A-Lyzer MWCO 10,000 G2 dialysis cassette (Thermo Fisher Scientific, Waltham, MA, USA). The control condition contained 680 μL of MMAF@BBN-PEG-PPADT nanoparticle-containing solution and 2789.2 μL PBS. The dialysis cassette was incubated in 100 mL PBS with 30 rpm magnetic stirring. At predetermined time intervals, 21 μL samples in the dialysis cassette were taken out and was replaced with fresh 21 μL PBS buffer. The acquired samples were mixed with 9 μL acetonitrile, and then analyzed by HPLC to quantify the amounts of MMAF.

### 2.7. In Vitro Cellular-Uptake Test With CLSM

Human prostate cancer cells (PC-3, American Type Culture Collection) were cultured in RPMI medium containing 10% FBS and 1% penicillin/streptomycin. Cells were grown and maintained in a humidified atmosphere with 5% CO_2_ at 37 °C. Cells were seeded onto 8-well plates at a density of 1 × 10^4^ cells/well on the LabTek II coverglass (Nalge Nunc International, Rochester, NY, USA). After 24 h, cells were treated with the nanoparticles and incubated for 4 h. For the competitive inhibition assay, prior to the treatments of the nanoparticles, free BBN peptide was pretreated to the cells at final concentrations of 0.1 mg/mL and incubated for 30 min. Cells were washed with DPBS three times and fixed with 4% para formaldehyde solution at room temperature for 1 h. The fixed cells were mounted by mounting medium containing DAPI, and the corresponding images were acquired by a CLSM (FLUOVIEW FV10i, Olympus).

### 2.8. In Vitro Cell-Cytotoxicity Test

The cytotoxicity was evaluated by the AlamarBlue assay. In brief, cells were seeded onto 96-well plates at a density of 2 × 10^3^ cells/well. After 24 h, each sample (free MMAF, MMAF@mPEG-PPADT, MMAF@BBN-PEG-PPADT) was treated in the cells in a dose-dependent manner. After 96 h incubation, each well was washed with DPBS and then 200 μL of fresh medium containing 10 μL of AlamarBlue agents was added to each well. After 1 h, the fluorescence (excitation 540 nm, emission 590 nm) was measured by using a microplate spectrofluorometer (BioTek Instruments, Winooski, VT, USA). The nontreated cells were used to represent 100% cell viability. The results are presented as mean ± standard deviation (*n* = 3).

### 2.9. Statistics

All experiments were repeated at least three times and each condition was analyzed in triplicate. The statistical significance of differences between experimental and control groups was determined using a Student’s *t*-test. Significant differences are denoted by asterisks in the figures.

## 3. Results and Discussion

### 3.1. Preparation of MMAF@BBN-PEG-PPADT

**Figure 1:**
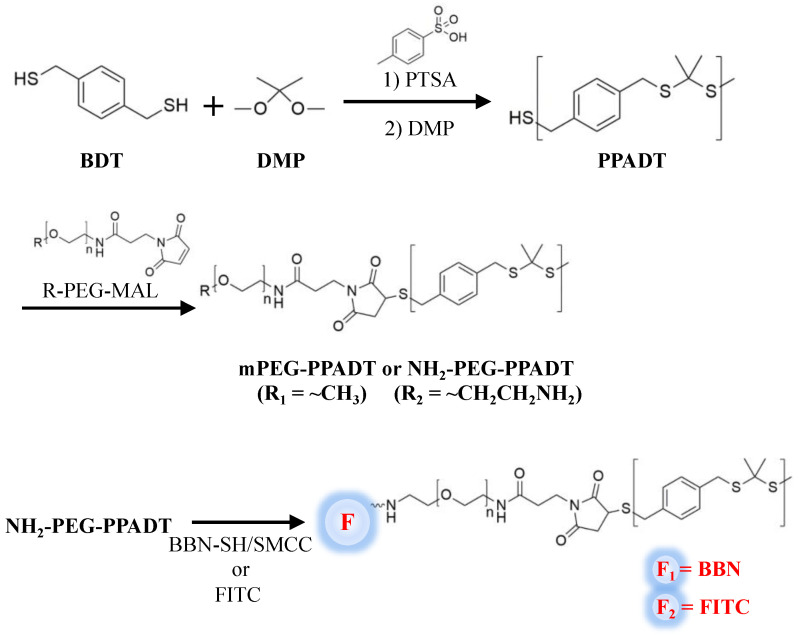
Synthetic scheme for mPEG-PPADT and BBN-PEG-PPADT.

**Figure 2:**
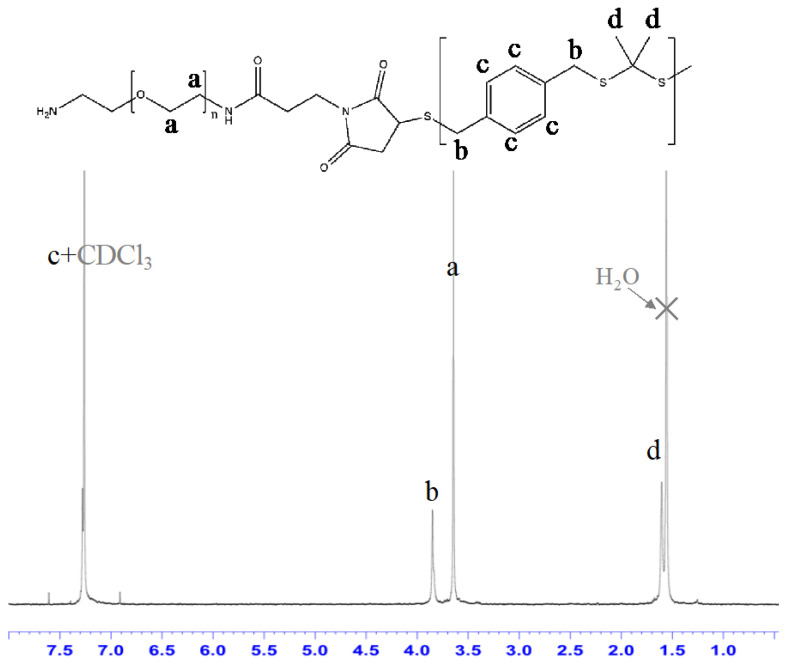
Characterizations of block copolymers, and ^1^H NMR of NH_2_-PEG-PPADT.

The PEG-modified PPADTs (mPEG-PPADT or NH_2_-PEG-PPADT) were synthesized according to the synthetic scheme as shown in Figure 1. DMP and BDT underwent condensation polymerization via acid-catalyzed acetal exchange reactions to afford the PPADT polymers, and then the maleimide functionalized PEGs (mPEG5K-MAL or MAL-PEG5K-NH_2_) were conjugated to the thiol groups of PPADT to afford the PEG-*b*-PPADT block copolymers (mPEG-PPADT or NH_2_-PEG-PPADT). The successful synthesis of NH_2_-PEG-PPADT was investigated by ^1^H NMR by confirming the characteristic peaks of benzene at *δ* 7.2 ppm, Ph-CH_2_-S- at *δ* 3.9 ppm, PEG at *δ* 3.7 ppm, and methyl at *δ* 1.6 ppm (Figure 2) by comparing with the ^1^H NMR of PPADT reported by J. S. Kim et al. [22]. The relative integration value of the PEG peak (*δ* 3.7 ppm) compared to that of the methyl peak (*δ* 1.6 ppm) of PPADT was used for the calculation of the molecular weight (Mn) of NH_2_-PEG-PPADT (Mn ≈12,300). BBN-tethered PEG-*b*-PPADT (BBN-PEG-PPADT) was synthesized by conjugating thiol groups of BBN to the amine groups of NH_2_-PEG-PPADT via SMCC crosslinker.

**Figure 3:**
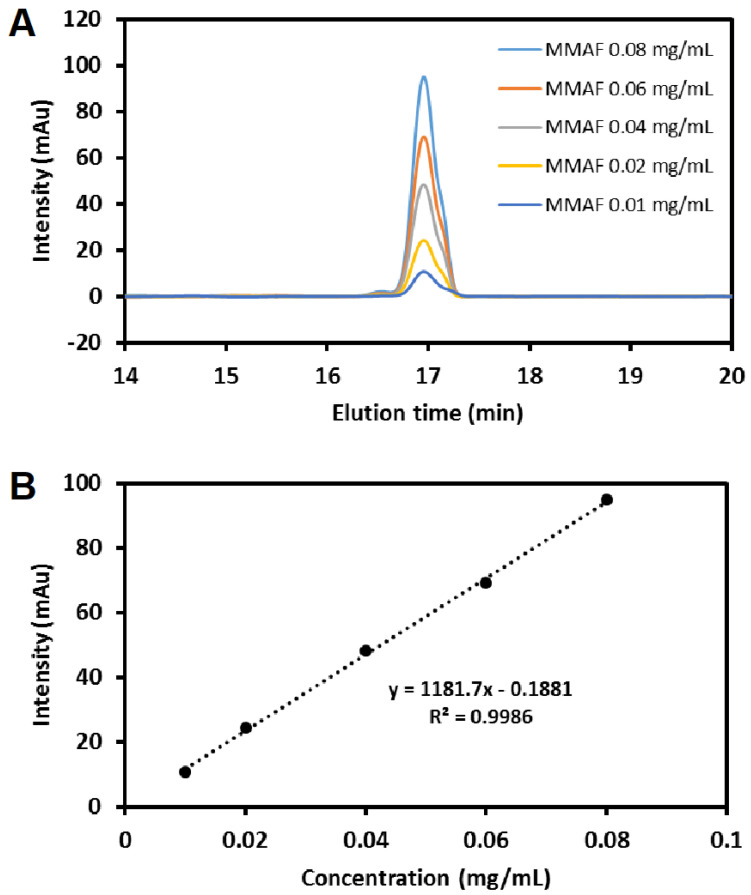
Quantification of MMAF using HPLC. (**A**) Concentration-dependent HPLC spectrum. (**B**) Standard curve of MMAF.

The MMAF-loaded BBN-PEG-PPADT nanoparticles (MMAF@BBN-PEG-PPADT) were prepared by hydrophobic interactions between benzene groups in PPADT and MMAF. The amounts of MMAF in the MMAF@BBN-PEG-PPADT were determined by reverse-phase HPLC (RP-HPLC) (Figure 3). The loading contents were 12.16 ± 0.64 wt %, and the loading efficiency was 91.29 ± 0.01%.

### 3.2. Characterization of MMAF@BBN-PEG-PPADT

**Figure 4:**
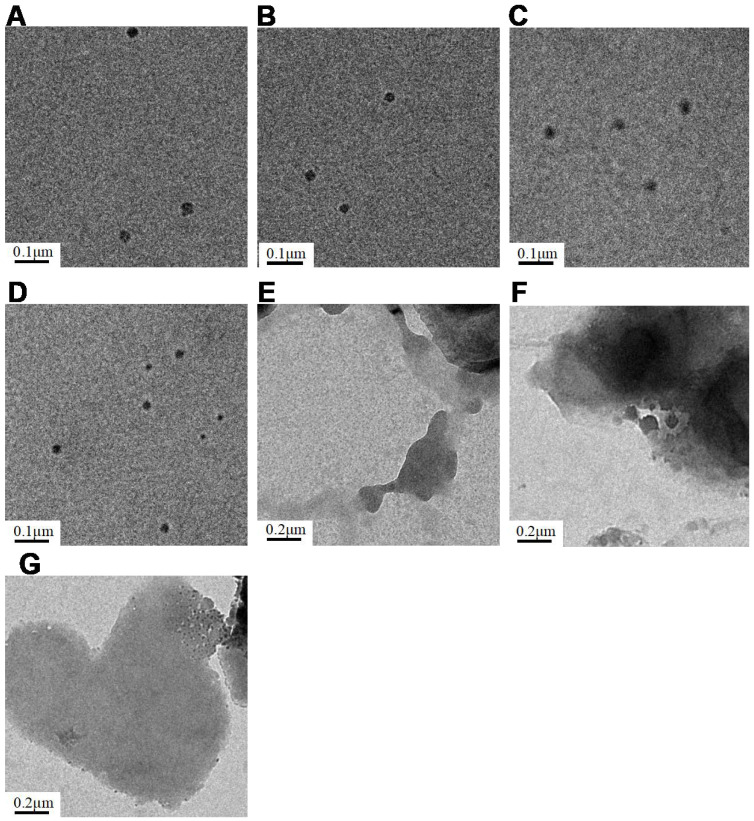
TEM images of MMAF@BBN-PEG-PPADT after (**A**) 0 h, (**B**) 1 h, (**C**) 4 h, and (**D**) 24 h incubation in PBS; and after (**E**) 1 h, (**F**) 4 h, and (**G**) 24 h exposure to ROS conditions.

**Figure 5:**
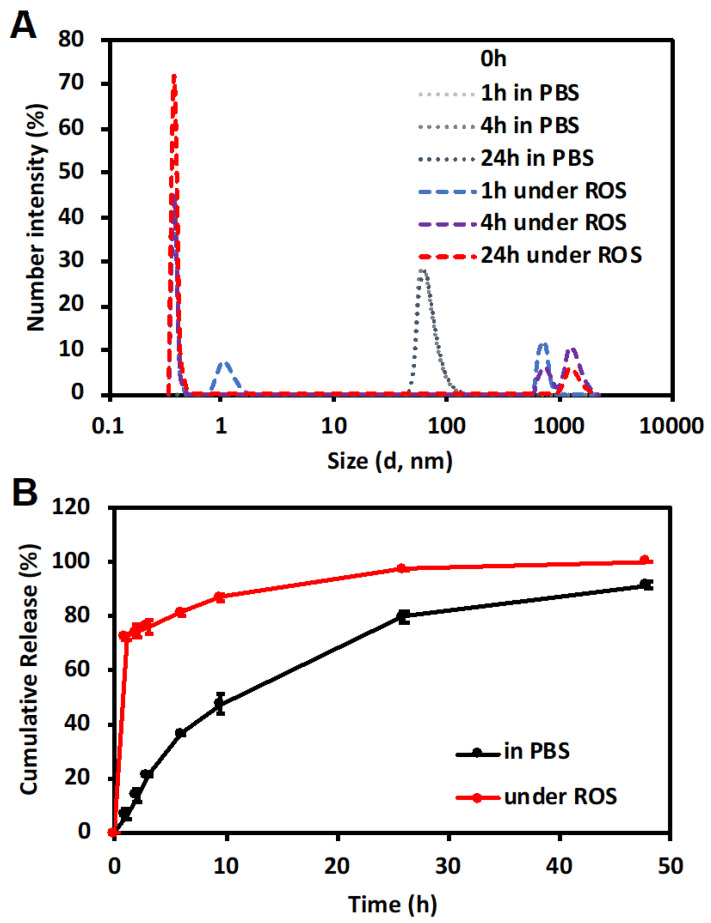
ROS responsiveness. (**A**) DLS of MMAF@BBN-PEG-PPADT before and after ROS exposure. (**B**) Drug-release behavior of MMAF@BBN-PEG-PPADT in PBS or under ROS.

The ROS-responsive behaviors of MMAF@BBN-PEG-PPADT were corroborated by TEM images and DLS. As shown in Figure 4A–D, the nanoparticles had spherical shapes whose morphology and size were retained even after 48 h incubation in PBS buffer. However, the nanoparticles were disrupted and formed large aggregates even after 1 h exposure to ROS conditions (Figure 4E–G). The DLS showed results similar to the TEM images (Figure 5A). The size of the nanoparticles was retained before (69.6 ± 2.0 nm) and after 24 h incubation (69.5 ± 0.7 nm) in physiological conditions (PBS buffer). However, it was changed to the large aggregates (744.7 ± 34.8 nm, 1383.2 ± 60.7 nm) and small molecules with a size around ~1 nm when the nanoparticles were incubated under ROS conditions. In particular, it appeared that the small molecules with a size of about 0.4 ± 0.2 nm accounted for the majority of the components under ROS conditions. However, it was due to the intrinsic limitations of the DLS techniques, which cannot measure the size of nondispersible large aggregates. Taken together, the TEM and DLS results suggested that the nanoparticles were stable in physiological conditions, whereas they were easily disrupted under ROS conditions to afford unmeasurable large aggregates and small molecules. These results may be ascribed to the disassembly of the nanoparticles through the ROS-responsive degradation of the hydrophobic PPADT polymers in BBN-PEG-PPADT.

The drug-release behavior of the nanoparticles was investigated in PBS and under ROS conditions, and the resultant cumulative release of MMAF versus time is shown in Figure 5B. The acquired standard curve (Figure 3B) was also used for determining drug release. MMAF@BBN-PEG-PPADT nanoparticles showed accelerated MMAF release under ROS conditions, whereas spontaneous MMAF release was demonstrated in physiological conditions. The half-life of the drug release clearly demonstrated the ROS-responsive, drug-releasing behaviors of MMAF@BBN-PEG-PPADT nanoparticles, as it was below 1 h under ROS conditions and 10.7 ± 1.6 h in the physiological conditions. Accordingly, we concluded that MMAF@BBN-PEG-PPADT nanoparticles showed ROS-responsive behaviors in terms of morphology and drug release.

### 3.3. In Vitro Cellular Uptake and Anticancer Efficacy of MMAF@BBN-PEG-PPADT

**Figure 6:**
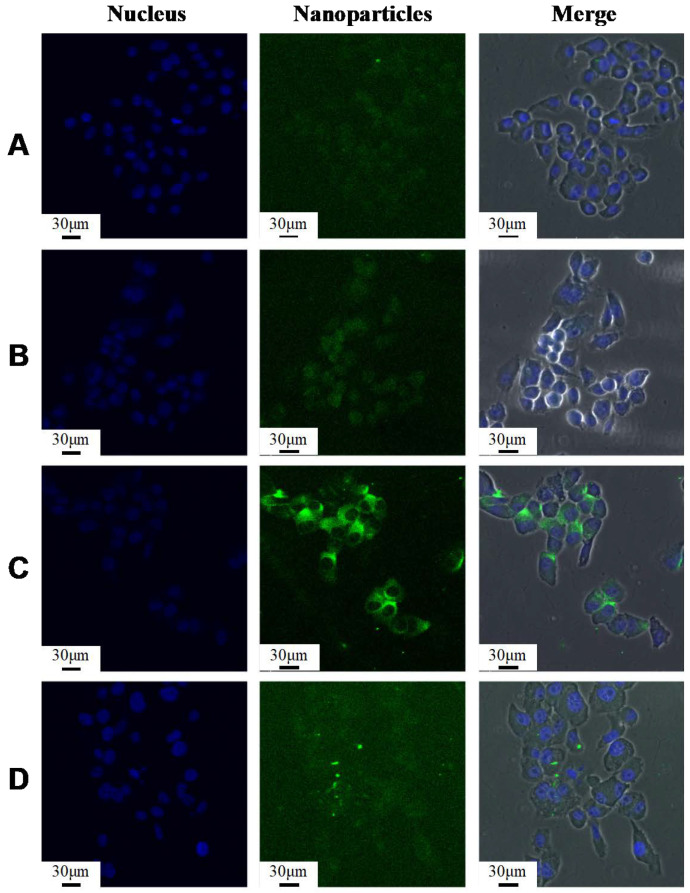
Confocal microscopy studies in PC-3 cells treated with (**A**) mPEG-PPADT, (**B**) mPEG-PPADT pretreated with free BBN, (**C**) BBN-PEG-PPADT, and (**D**) BBN-PEG-PPADT pretreated with BBN. Nuclei and nanoparticles were labeled with DAPI (blue) and FITC (green), respectively.

We expected that the BBN peptides would enhance the cellular uptake of the nanoparticles via receptor-mediated endocytosis. In order to investigate the ability of BBN for targeting and delivering MMAF to tumors, FITC-labeled MMAF@BBN-PEG-PPADT nanoparticles were prepared by mixing FITC-labeled PEG-PPADT (FITC-PEG-PPADT) in the preparation step of MMAF@BBN-PEG-PPADT nanoparticles. The PC-3 cell line was selected for the in vitro test, as it is well known to overexpress GRP receptors [17,18] as well as ROS [22,25]. MMAF@mPEG-PPADT was prepared and used as a control group. In addition, free BBN were pretreated with PC-3 cells for the competitive inhibition assay. As shown in the CLSM studies (Figure 6), predominant green fluorescence was observed in the cells incubated with MMAF@BBN-PEG-PPADT nanoparticles compared to MMAF@mPEG-PPADT nanoparticles. Furthermore, the cellular uptake of MMAF@BBN-PEG-PPADT nanoparticles was clearly reduced in the BBN-pretreated cells compared to nontreated cells, whereas the FITC signals of MMAF@mPEG-PPADT were insignificantly different between BBN-pretreated and nontreated cells. These results demonstrated that the BBN peptides played an essential role in the enhanced cellular uptake, implying the improved anticancer efficiency.

**Figure 7:**
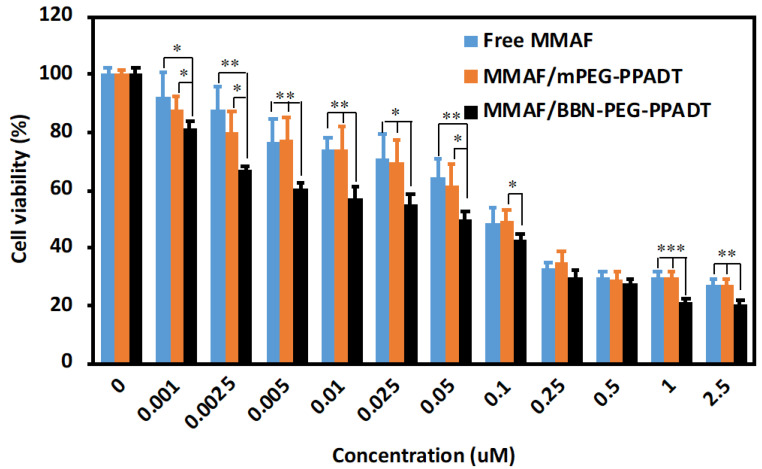
In vitro cell-viability test in the PC-3 cell line. *** *p* < 0.001, ** *p* < 0.01, and * *p* < 0.05, which were analyzed with Student’s *t*-test.

Finally, we investigated the anticancer effects of the MMAF-loaded nanoparticles. As shown in the AlamarBlue assay (Figure 7), MMAF@mPEG-PPADT exhibited similar cytotoxicity with free MMAF. Although PEG has been widely used for numerous drug-delivery systems due to its antifouling effects during blood circulation in vivo, it is also well known to inhibit the cellular uptake of nanoparticles. Accordingly, the free drug generally has higher cytotoxic effects than the drug-loaded nanoparticles in vitro [26,27]. In order to circumvent this intrinsic limitation of PEG, various intracellular stimuli-responsive drug-delivery systems have been developed, as they allow the increase of the intracellular level of anticancer drugs instantaneously and more than the cancer cells can withstand by facilitating the burst drug release. Therefore, the similar cytotoxic effects of MMAF@mPEG-PPADT with free MMAF were ascribed to the ROS-responsive burst drug-releasing behaviors. In particular, MMAF@BBN-PEG-PPADT showed higher anticancer effects than MMAF@mPEG-PPADT and free MMAF. This was attributed to the BBN-mediated enhanced cellular uptake of the nanoparticles.

In summary, MMAF has not been in clinical translation due to its intrinsic limitations in ADC formulations. Herein, efficient MMAF delivery was achieved by developing MMAF-loaded BBN-PEG-PPADT micelles. BBN peptides allowed the efficient intracellular uptake of nanoparticles into the targeted cancer. ROS-responsive degradation of PPADT facilitated the intracellular disruption of nanoparticles and followed burst release of MMAF. The increased cellular uptake and anticancer effects imply a promising potential of cancer-targeting, ROS-responsive nanoparticle systems in realizing an efficient delivery of MMAF.
